# TOXIC EXPOSURES IN CHILDREN INVOLVING LEGALLY AND ILLEGALLY COMMERCIALIZED HOUSEHOLD SANITIZERS

**DOI:** 10.1590/1984-0462/;2017;35;1;00010

**Published:** 2017

**Authors:** Alessandra Marcuz de Souza Campos, Fábio Bucaretchi, Luciane Cristina Rodrigues Fernandes, Carla Borrasca Fernandes, Eduardo Mello de Capitani, Ana Raquel Medeiros Beck

**Affiliations:** a Centro de Controle de Intoxicações da Faculdade de Ciências Médicas da Universidade Estadual de Campinas (UNICAMP), Campinas, SP, Brasil.; b Faculdade de Enfermagem da UNICAMP, Campinas, SP, Brasil.

**Keywords:** Poisoning, Sanitizing products, Household products, Caustics, Endoscopy, Child

## Abstract

**Objectives:**

To analyze and to compare clinical repercussions of accidents involving legally and illegally commercialized household sanitizers in children under 7 years of age.

**Methods:**

A descriptive cross-sectional design was used to collect data from electronic database of a regional Poison Control Center during one year. Data were analyzed by means of descriptive non-parametric statistics and association tests.

Results: The sample had 737 reported cases. Most of the accidents occurred with children under 3 years of age (median: 1 year of age; interquartile interval: 1-3 years of age), at home (92.9%), by ingestion (97.2%). Products involved were cleaning products with low toxicity and no caustic effects (38.9%); caustics (24.1%); hydrocarbons (19.3%); pesticides/rodenticides (16.6%), and other products (1.1%). Seventy accidents were due to exposures to illegal products, mainly caustics (n=47) and rodenticides (n=15). Among the 337 children presenting post-exposure clinical manifestations, the most frequent were vomiting (n=125), oral burns (n=74), cough (n=35), drooling (n=26), and abdominal pain (n=25). Clinical manifestations were significantly more frequent after illegal products exposure (55/70 versus 282/667, p<0.01). Nineteen children had to be hospitalized (caustics, n=17; illegal products, n=12; median time of hospitalization: 2 days), 22 were submitted to esophagogastroduodenoscopy (sodium hydroxide, n=14; illegal products, n=14); and 12 cases had endoscopic alterations (severe in 2). No deaths occurred.

**Conclusion:**

Toxic exposures owing to illegal household sanitizer products are associated with greater morbidity when compared with legal ones.

## RESUMO

**Objetivos:**

Analisar e comparar as repercussões clínicas dos acidentes com saneantes de uso domiciliar de origem legal e ilegal (clandestina) em crianças menores de 7 anos.

**Métodos:**

Estudo descritivo de corte transversal, com dados obtidos dos prontuários eletrônicos do Centro de Informações e Assistência Toxicológica de referência regional, no período de um ano completo. Foram realizadas análises estatísticas descritivas não paramétricas e de testes de associação. Resultados: A amostra foi constituída de 737 casos. A maioria das exposições ocorreu em crianças menores de 3 anos (mediana: 1 ano, intervalo interquartil: 1-3 anos) na residência habitual (92,9%) e por ingestão (97,2%). Os produtos envolvidos foram saneantes de baixa toxicidade sem efeito cáustico (38,9%), com efeito cáustico (24,1%), hidrocarbonetos (19,3%), inseticidas/raticidas (16,6%), e outros produtos (1,1%). Setenta casos decorreram de exposições a produtos clandestinos, principalmente cáusticos (n=47) e raticidas (n=15). Entre as 337 crianças que apresentaram manifestações clínicas pós-exposição, as ocorrências mais frequentes foram vômitos (n=125), queimaduras orais (n=74), tosse (n=35), salivação (n=26) e dor abdominal (n=25), significativamente mais comum com produtos clandestinos (55/70 versus 282/667; p<0,01). Dezenove crianças foram hospitalizadas (cáusticos, n=17; produtos clandestinos, n=12; mediana do tempo de internação: 2 dias), e 22 foram submetidas à endoscopia digestiva alta (hidróxido de sódio, n=14; produtos clandestinos, n=14), com alterações em 12 casos (grave=2). Não houve óbitos.

**Conclusões:**

Exposições tóxicas a saneantes de uso domiciliar de origem clandestina estão associadas com maior morbidade quando comparadas aos de venda autorizada.

## INTRODUCTION

Toxic exposures to free sale sanitizers in children are common and have high rates of morbidity, especially those involving caustic and hydrocarbon.1-3 To make matters worse, in Brazil, a large portion of the population also uses and stores unauthorized cleaning products for household consumption, named “illegal” or “clandestine.”^4^ ^,^ ^5^

Clandestine sanitizers of unauthorized manufacturing are formulations sold without registration in the National Health Surveillance Agency (ANVISA).^6^ For a sanitizer to receive approval for registration at ANVISA various items such as risk management, usage, and category are analyzed. In the assessment and risk management, the toxicity of substances and their concentrations in the product, the purpose and conditions of use, the occurrence of adverse events or previous technical complaints, the likely exposed populations, the frequency of exposure and the duration, and forms of preparation are considered.

In addition, companies legally authorized to manufacture, store, distribute, transport, fractionate, or import sanitizing products are subject to verification of compliance with “Good Manufacturing Practices and Control.”^6^ Illegal products are usually sold by street vendors in a door-to-door approach, although they can also be found in general cleaning products stores, including public markets.^5^ ^,^ ^7^ ^,^ ^8^ A study that examined 419 urban households in the federal capital showed that of the 239 homes where children lived, 30.1% stored illegal sanitizers, leading to a potential risk of accidental toxic exposures to these products.^4^

Illegal sanitizers most often have colors that are very attractive to children and are usually stored in reused packages of soft drinks, in two-liter bottles, commonly denominated in Brazil “roxinho (in *English, “purple*”).”^1^ ^,^ ^4^ ^,^ ^5^ Furthermore, in most cases, the label containing product formulation is missing in the package of these illegal products or, if a label is attached, its information is usually incorrect or false.^1^ ^,^ ^4^ ^,^ ^5^

In view of these considerations, the objective of this study was to analyze and compare the clinical consequences of accidents with legal and illegal household sanitizers in children aged below 7 years.

## METHOD

This is a descriptive cross-sectional study. Data were collected from health care records of the Information and Toxicological Assistance Centre of Campinas (CIATOX), which functions as a Poison Control Center (PCC) and is a reference service of the administrative region of Campinas, in the state of São Paulo, in southeastern Brazil. This administrative region encompasses 90 municipalities and population estimated at 6.5 million of inhabitants. Since October 2013, the assistance of CIATOX in Campinas has been recorded in real time in the electronic base of the Brazilian Information System on Intoxication (DATATOX) of the Brazilian Association of CIATOX (ABRACIT), which generates a database and electronic records containing all information collected.

All patients under 7 years of age, who were accidentally exposed to household sanitizers that were sold legally and illegally, were considered eligible. The information was collected in a full year period (1 October 2013 to 30 September 2014) and refers to cases assisted and monitored by telephone or in person (patients admitted to the Pediatric Emergency Sector of the *Hospital das Clínicas da Universidade Estadual de Campinas* - Unicamp).

For purpose of analysis, the products were arbitrarily divided into five classes:

1. low toxicity sanitizers, such as bleach for laundry and general use disinfectants that do not contain chlorine in its formulation, detergents for dish wash, soap powders or bars, fabric softeners, multipurpose cleaners, and home fragrances;
2. sanitizers with caustic effect that include sodium hypochlorite and sodium hydroxide on their formulations, “*roxinhos*,” products with formulations based on chlorine for pool treatment, degreasers, descaling products, and acid or alkaline products for cleaning aluminum, stones, or ovens;
3. hydrocarbon-based sanitizers, such as removers, kerosene, turpentine, paint thinner, cresols, pine oils, and waxes;
4. insecticides and rodenticides, as pyrethroids, naphthalenes, formicides, roach killers, and legal (coumarin) and illegal (aldicarb/carbofuran - known as “*chumbinho*”) rodenticides;
5. others, such as sanitizers whose composition was unclear and could not be classified according to the four previous categories. Rodenticides named “chumbinho”[Bibr B1] are usually produced using cholinesterase inhibitors, particularly carbamates such as aldicarb and carbofuran.^8^

If the child was exposed to more than one product, the product with the greatest toxicity was considered. Exposures to caustic products were also analyzed separately, owing to their greater morbidity.

Data for each case were inputted into a spreadsheet built for the study (Excel, Microsoft Office^®^ 2010). Descriptive and nonparametric statistical analyses were performed [median and interquartile range (IQR)] and association tests (chi-square and Fisher’s exact test) as appropriate, adopting as significance level a p-value of ≤0.05.

The study was approved by the Ethics Committee of the School of Medical Sciences of Unicamp, under the opinion number 853,646 and CAAE number 37346214.8.0000.5404.

## RESULTS

The sample consisted of 737 cases - 15.3% of all cases monitored by the CIATOX during the study period. Most of the assistance was carried out only by phone (98.1%), covering information requested by the physician (64.4%), by the hospital services (56.7%), followed by telephone calls from parents/relatives made from their homes (31.9%). Assistance requests were mainly generated from the administrative region of Campinas (67.3%).

Most of the exposures occurred in children below 3 years of age (79.6%; median=1 year, IQR 1 to 3 years, limits of 57 days to 6 years), at home (92.9%), and with slight predominance of males (55.6%). With regard to the routes of exposure, the main route was ingestion (97.2%), followed by skin absorption (6.6%). Simultaneous routes of exposure were found in 52 cases (two routes, n=48; 3 routes, n=4). Table 1 contains the main demographic characteristics, routes of exposure, and the evolution of the exposed population, according to the five classes of products.

**Table 1:**
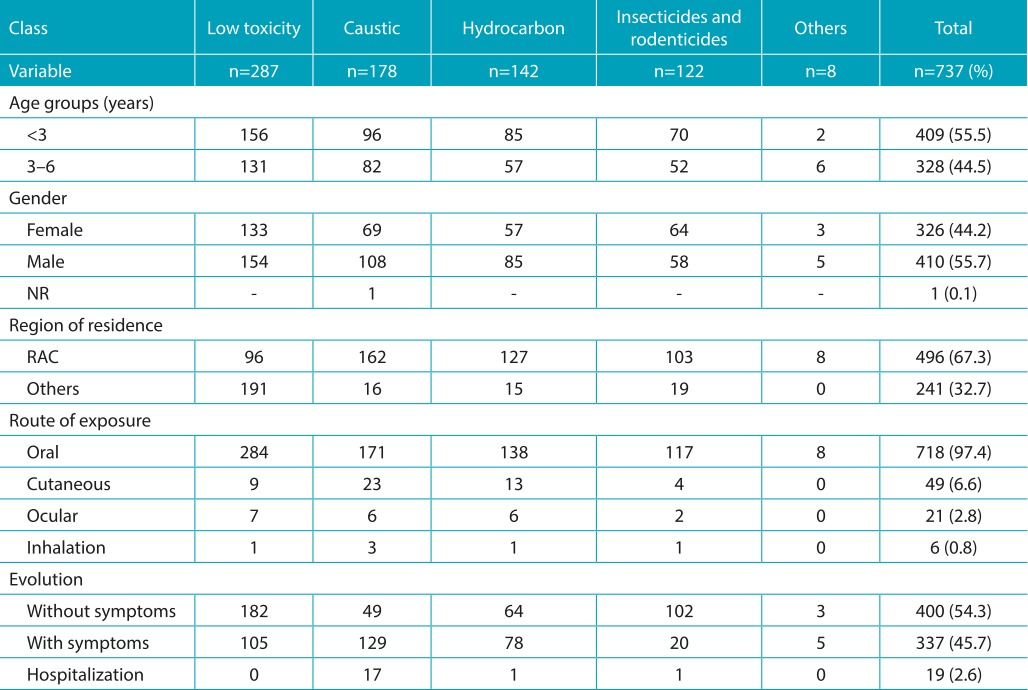
Demographic characteristics, routes of exposure, and clinical evolution of the studied population, according to according to the class of the product.

NR: not reported; RAC: administrative region of Campinas; Route of exposure: 52 patients were simultaneously exposed to more than one route.

Low toxicity sanitizers (38.9%) and caustic products (24.1%) were the main sources of exposure. It was also found that 9.5% of the exposures were caused by illegal products, especially caustic products and rodenticides (Table 2). Exposure to products manufactured in the household was recorded in 12 cases, especially to “homemade soap” (n=10), which contains sodium hydroxide (caustic soda) used in the manufacturing process. It is worth mentioning that all ten children exposed to this type of product developed postexposure clinical manifestations.

**Table 2:**
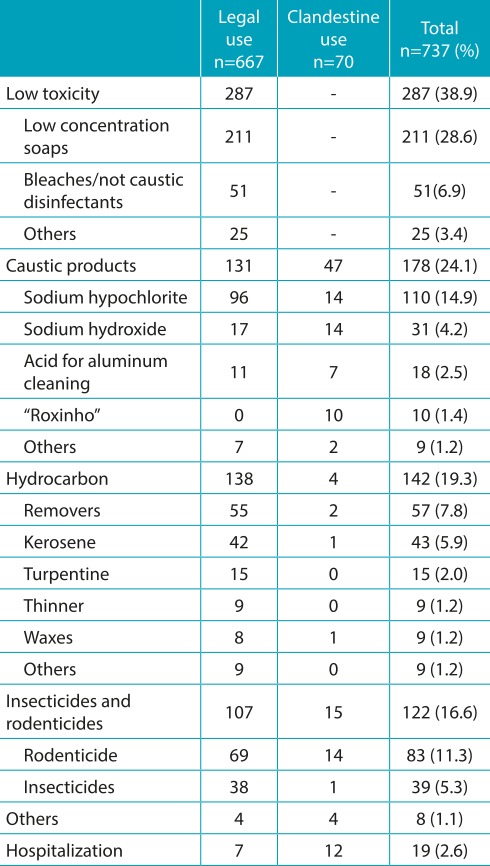
Frequency distribution of the agents involved by class and the origin of the product (legal or illegal use).

With regard to actions taken prior to the contact with CIATOX, at home the relatives offered milk, other liquids, and foods to the child in 76 cases, of which 37 had ingested caustic products (sodium hydroxide, n=13, sodium hypochlorite, n=16; “*roxinho*”, n=4; others, n=4) and 11 had ingested hydrocarbons; 28 evolved to vomiting, with more than one episode in 14 cases. In 11 patients vomiting was also induced at home, six of which ingested caustic substances (sodium hypochlorite, n=3; acids for cleaning aluminum, n=2; “*roxinho*”, n=1) and one ingested hydrocarbon. Gastric lavage was performed in health services in 25 cases (3.4% of the total), of which 14 children ingested rodenticides (coumarin, n=10; “*chumbinho*”, n=3; product not determined, n=1), 7 children ingested caustic substances (sodium hypochlorite, n=6; acid to clean aluminum, n=1), 3 ingested hydrocarbons (removers, n=2, kerosene, n=1), and one ingested an illegal carbamate insecticide of clandestine use (methomyl).

Among the 337 children who presented postexposure clinical manifestations, the most common were vomiting (n=125), oral burns (n=74), cough (n=35), salivation (n=26), and abdominal pain (n=25). These manifestations were significantly more common in exposures to illegal products (55/70 *versus* 282/667; p<0.01). With regard to the 35 patients who developed cough, 21 were exposed to hydrocarbons, of which 14 also presented vomiting. Regarding the 14 children exposed to illegal rodenticides, 12 ingested “*chumbinho*”; only one needed an antidote (atropine). Table 3 contains the main clinical manifestations detected and the association of the product types (legal or illegal) with the occurrence of clinical manifestations and the need for hospitalization.

**Table 3:**
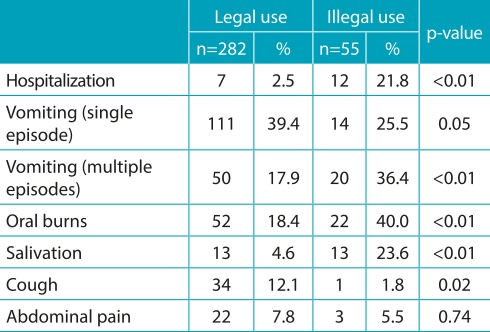
Association of exposure to legal and illegal cleaning products for domestic use in 337 children with postexposure symptoms, such as the evolution for hospitalization and type of clinical manifestation reported.

Considering the 178 children exposed to caustic products, 129 (72.5%) presented clinical manifestations in the following proportions according to the type of product: sodium hydroxide, 27/31 (87.1%); sodium hypochlorite, 74/110 (67.3%); “*roxinho*,” 10/10; and other caustic products, 18/27 (66.7%). Clandestine caustic products were significantly associated with higher incidence of hospitalization compared to legal products (11/43 *versus* 6/86; p<0.01) as well as with the need for upper gastrointestinal endoscopy (14/43 *versus* 8/86; p<0.01).

Most of the 19 children who required hospitalization had short-duration hospital stay (median=2 days, IQR, 1-3 days), resulting from exposure the caustic products (sodium hydroxide, n=12; “*roxinho*,” n=3; sodium hypochlorite, n=1; other, n=1), followed by one case of exposure to hydrocarbon and one case of exposure to “*chumbinho*.” Three male patients needed longer hospital stay as follows: two owing to serious esophagitis after the ingestion of sodium hydroxide (10 months and 6 years of age, and length of stay of 9 and 45 days, respectively); and 01 male patient aged 1 year, owing to the ingestion of hydrocarbon (remover of legal use). This patient evolved to a probable diffuse alveolar damage (chemical pneumonitis) with respiratory difficulties, requiring supplemental oxygen (seven days of hospitalization). No death was reported.

Table 4 contains information concerning the products involved, the clinical manifestations, and endoscopic alterations found in 22 patients undergoing this procedure. In only two cases, endoscopic Zargar’s grading classification (I to III) was applied to the cases of chemical burns caused by caustic products,^9^ described as I (edema and hyperemia of the mucosa without ulcers) and IIa (submucosal lesions, ulcers, or exudates without circumferential esophageal injury), in that order.

**Table 4:**
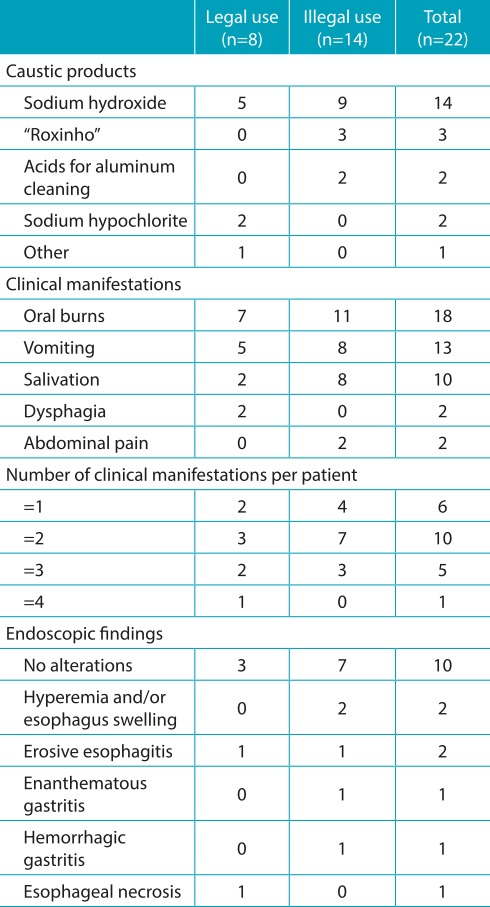
Caustic products involved, clinical manifestations, and endoscopic alterations found in 22 patients undergoing upper gastrointestinal endoscopy.

## DISCUSSION

The study results revealed an epidemiological overview of exposure to legal and illegal household sanitizers in children, in an important region of the state of São Paulo. The largest proportion of exposures in children below 3 years of age, male gender, and at home are consistent with the pattern observed in other studies^1^ ^,^ ^2^ ^,^ ^3^ ^,^ ^9^ ^,^ ^10^ and are associated with the child development stage characterized by the inability to recognize risks, the natural curiosity, the independent mobility with exploratory behavior, the proximity to the floor, and the habit of taking objects to mouth very often.^1^ ^,^ ^2^ ^,^ ^3^ ^,^ ^10^ In addition to these observations, the results confirm a greater number of hospitalizations resulting from exposure to caustic products, similar to that found in the US pediatric emergency services.^3^

Considering the decontamination procedures still carried out at home and in the health services, several ineffective procedures or even iatrogenic effects were identified.^1^ ^,^ ^2^ ^,^ ^11^ ^,^ ^12^ Gastric lavage and inducing vomiting are formally contraindicated for patients who ingested caustic substances or hydrocarbons, owing to the risk of worsening chemical burns and developing chemical pneumonitis by aspiration, respectively.^2^ ^,^ ^11^ ^,^ ^12^ Furthermore, the recommendation of gastric lavage is progressively in disuse, with extremely limited indications, even in intentional intakes^12^ and should follow a previous discussion between the health professional and specialized health service, for example, the regional CIATOX.

Exposure to illegal products represented 9.5% of the total sample; however, it was associated with increased morbidity, directly related to the types of product involved (caustic), including increased number of children undergoing upper gastrointestinal endoscopy and hospitalizations. With regard to exposure to illegal rodenticide “*chumbinho*,” just one child of 12 children evolved with clinical manifestations of a cholinergic syndrome and needed to use atropine. These data suggest a possible reflection of the removal of aldicarb (Temik 150) of the Brazilian market since the end of 2012.^13^

The occurrence of postexposure clinical manifestations as risks markers for evolution to serious esophageal injury is still a matter of debate.^14^ ^,^ ^15^ ^,^ ^16^ ^,^ ^17^ In general, in unintentional ingestions of caustic substances, endoscopy should be performed in all patients with stridor and in any patient who evolve with two or more symptoms such as vomiting, salivation, and pain.^15^

Endoscopic findings as circumferential lesions of the submucosa, with presence of ulcers and exudates (classification IIb), or deep ulcers with necrosis of esophageal tissue layers (classification III), are important predictive risk markers of evolution to esophageal stricture scar.^9^ ^,^ ^16^ ^,^ ^17^ Thus, early endoscopy, between 12 and 24 hours after exposure in patients with indicative clinical manifestations of caustic injury has prognostic value and assists in indicating a more appropriate therapy in serious cases, such as placement of intraluminal stents and nasogastric tubes, aiming at preventing or minimizing the development for esophageal stenosis in patients at risk.^16^ ^,^ ^17^ In this series, in only two children among those 22 undergoing endoscopy the Zargar grading was applied.^9^ The relevance of the use of this classification in the issuance of reports should be emphasized to the endoscopists in the region.

Multicenter study conducted in Italy in 2008 showed a correlation of the presence of clinical manifestations (oral/oropharynx burns, vomiting, dyspnea, dysphagia, salivation, and hematemesis) with progression to serious esophageal burns (Zargar III) in 162 children, after accidental ingestion of caustic substances, and revealed that patients with one, two, three, or more signs/symptoms showed evolution of odds ratios for serious esophageal injury of 7.71, 6.69, and 11.97, respectively.^17^

In our series, patients who developed esophageal necrosis presented with four signs/symptoms on hospital admission, such as oral burns, several episodes of vomiting, dysphagia, and abdominal pain after ingestion of caustic soda that was stored in a soft drink bottle. In contrast, no endoscopic abnormality was detected in five patients undergoing endoscopy who showed only one sign/symptom - four with isolated oral burns and one with several episodes of vomiting.

The use of packages with child-proof caps is a preventive measure which proved to be effective in reducing child mortality by poisoning.^1^ ^,^ ^18^ ^,^ ^19^ Therefore, in 2013, ANVISA published a resolution that requires rigid tear-resistant plastic packing, with double safety child-proof cap for corrosive products.^20^ This regulation came into force in the same period the data collection for this study was initiated; therefore, it was not possible to assess its effect. However, as recommended in other countries, we understand that security packaging should be mandatory not only for corrosive products but also for drugs, other sanitizers, hydrocarbons, and pesticides.^1^ ^,^ ^2^ ^,^ ^18^ ^,^ ^19^ As recent tragic example, we had the case of a 1-year boy assisted in early 2015 and monitored by our CIATOX who died after accidental ingestion of a legal pyrethroid insecticide. The death was due, however, to the cardiotoxicity and pulmonary injury caused by high concentration of aliphatic hydrocarbons contained in the formulation (~97%), which was identified in the patient blood sample collected prior to the death (gas chromatography-mass spectrometry).

In addition to the aforementioned preventive measures, others need to be implemented, such as ongoing educational activities including sharing information through mass media concerning the importance of storing sanitizers and other products in safe places and out of child’s reach, the risks related to the consumption of illegal products, including those made in the household, such as homemade soaps as well as the wider dissemination of the activities of the Brazilian CIATOX for health professionals and general population.^1^

In conclusion, despite the limitations of a retrospective analysis, the results can be useful for preventing and improving the assistance to the pediatric population exposed to household sanitizing products, which shows higher morbidity in exposures to illegal products. Furthermore, the results confirm the relevance of the CIATOX in the assistance support to the population on toxicological emergencies and toxicovigilance.^1^ Therefore, in 2015, the Brazilian Ministry of Health recognized the CIATOX as health establishments that are members of emergency attention network of the Brazilian Unified Health System (SUS).^21^
